# Skin Inflammation Modulation via TNF-α, IL-17, and IL-12 Family Inhibitors Therapy and Cancer Control in Patients with Psoriasis

**DOI:** 10.3390/ijms23095198

**Published:** 2022-05-06

**Authors:** Daniel Octavian Costache, Oana Feroiu, Adelina Ghilencea, Mihaela Georgescu, Ana Căruntu, Constantin Căruntu, Sorin George Țiplica, Mariana Jinga, Raluca Simona Costache

**Affiliations:** 1 2nd Dermatology Discipline, Faculty of Medicine, Carol Davila University of Medicine and Pharmacy, 050474 Bucharest, Romania; daniel.costache@umfcd.ro (D.O.C.); gstcolentina@gmail.com (S.G.Ț.); 2 Research Department, Carol Davila University Central Emergency Military Hospital, 010242 Bucharest, Romania; 3 Dermatology Department, Carol Davila University Central Emergency Military Hospital, 010242 Bucharest, Romania; dabeleaoana@gmail.com (O.F.); adelina.ghilencea@yahoo.com (A.G.); georgescucmihaela@gmail.com (M.G.); 4 Faculty of Medicine, Titu Maiorescu University, 040441 Bucharest, Romania; ana.caruntu@gmail.com; 5 Oral and Maxillofacial Surgery Department, Carol Davila University Central Emergency Military Hospital, 010242 Bucharest, Romania; 6 Physiology Discipline, Carol Davila University of Medicine and Pharmacy, 050474 Bucharest, Romania; costin.caruntu@gmail.com; 7 Internal Medicine and Gastroenterology Discipline, Faculty of Medicine, Carol Davila University of Medicine and Pharmacy, 050474 Bucharest, Romania; mariana_jinga@yahoo.com

**Keywords:** systemic inflammatory syndrome, psoriasis, cancer, environment-inadequacy status, biologic therapy

## Abstract

The systemic inflammatory syndrome concept is one of the foundations that stand at the basis of revolutionary modern and future therapies, based on the in-depth understanding of the delicate mechanisms that govern the collaboration between the systems and organs of the human body and, at the same time, the fine balance that ensures a reproach-free operation. An interesting concept that we propose is that of the environment-inadequacy status, a concept that non-specifically incorporates all the situations of the organism’s response disorders in the face of imprecisely defined situations of the environment. The correlation between these two concepts will define the future of modern medicine, along with the gene-adjustment mechanisms. Psoriasis is a clear example of an inadequate body response as a result of exposure to as of yet undefined triggers with an excessive systemic inflammatory reaction and hitherto insufficiently controllable. Modern biological therapies, such as TNF-α, IL-12 family, and IL-17 inhibitors, are intended to profoundly reshape the cytokine configuration of patients with inflammatory diseases such as psoriasis, with tremendous success in disease control. Yet, because of the important roles of cytokines in cancer promotion and control, concern was raised about the fact that the use of biologicals may alter immune surveillance and promote cancer progression. Both theoretical and practical data nevertheless showed that the treatment-induced control of cytokines may be beneficial for reducing the inflammatory milieu that promotes cancer and such have a beneficial role in maintaining health. We briefly present the intricate roles of those cytokine families on cancer control, with some debates on if their inhibition might or might not promote additional tumoral development.

## 1. Introduction

Psoriasis is a chronic papulosquamous [1,2], immune-mediated [3] inflammatory disease of unknown etiology, with genetic predisposition [4], which affects mainly the skin and articulations [5], but which associates serious comorbidities, being a systemic disorder [6].

Psoriatic arthritis is a chronic inflammatory disease [7], part of the spondyloarthropathy class, which may appear concomitantly with psoriasis in a significant number of patients [8].

Nowadays, we frequently encounter the association of psoriasis with metabolic syndrome, inflammatory bowel conditions, depression, or heart disease, the common point of which is uncontrolled systemic inflammation triggered by an unknown cause [9,10,11,12,13]. Modern biologic molecules therapy defines a new perspective for these patients, with a superior success rate and stable control of clinical manifestations and systemic inflammation. Yet, possible adverse effects of biological treatments as immune modulators maintain a rising level of consciousness regarding the possibility of infections and neoplasia in the patients.

The pathogenic mechanisms are yet imprecisely known. They are considered to be defined by impaired immune surveillance and a specific association of comorbidities, amongst which cancer plays a significant role because of its prognosis. Whether there is a definitive specific association between psoriasis and cancer remains yet to be proved. The association of habits such as alcohol and tobacco consumption and the chronic therapeutic exposure to UV light seems to play a role in the increased risk of neoplasia—it is to discuss if they are independent risk factors or if the correlation is somehow related to the presence of psoriasis, even if the additional cancer risk given by the association of psoriasis could be only described as small [14,15]. Even more, if there is a somewhat clear association between psoriasis and non-melanoma skin cancers (NMSC)—considered to be conditioned by the specific therapies—the risk of solid organ malignancies seems to be no higher than in the general population [16]. The increased association between psoriasis and lung cancers might need an adjustment for smoking habits [16].

Given the involvement of the inflammatory system in the control of cell multiplication in the body, the discussion about the correlations between chronic systemic inflammatory syndrome and hematological or organ tumors is natural. Thus, in terms of chronic psoriasis vulgaris, the significant role of the increased tissue and blood titers of TNF-α in the appearance of clinical manifestations of the disease is demonstrated. As such, therapeutic formulas have been developed that reduce TNF-α concentrations, managing a good and very good control of the disease but bringing in parallel a series of side effects [11,17].

In patients with psoriasis, TNF-α, IL-12 family, and IL-17 cytokines were selected as the main therapeutic target of the new drugs.

We started using TNF-α inhibitors in 2005, and from then, several hundred patients were treated by our team. More recently, interleukin inhibitors became a new therapeutic tool with better results. Most of our patients were over 50 years old, and concerns about their overall health status represented an important aspect. TB and neoplasia screenings were precedent in our patients’ management.

Many papers presented over time in national and international congresses pointed out the risk of neoplasia in biologic therapy patients. Searching on PubMed, over 27,000 papers discuss biological therapy and cancer; of those, almost 13,000 were published in the last 10 years, and 7675 in the last five years. In total, 470 reviews and 33 systematic reviews were published in the last year. Important details may be found in the nine reviews published in the last 5 years concerning psoriasis, biological treatment, and cancer (such as [18,19]).

All this amount of professional literature underlines the importance of the subject. For those who would like to deeper study the matter, a wide variety of information is available. It is not our intention to propose a new review on the matter; instead, we present our perspective as a result of our concerns and thoughts, as they resulted after years of experience.

## 2. TNF-α

TNF-α is a well-known molecule, discovered more than 40 years ago (1975), having extensive roles in immunity and inflammation, apoptosis, and cell survival control. Even the name speaks loud about its roles; it started with the proof of hemorrhagic necrosis on mice tumors (Coley’s toxin) [20]. Initially, it was also named cachectin and T-lymphocyte differentiation factor [21].

At present, it is proven that TNF-α plays an important role in the pathogenesis of psoriasis, and because of that, it has become the first target of modern therapies. For the first time, the use of TNF-α inhibitors offers a disease control that gives hope for a better life for patients with severe, difficult to control diseases.

TNF-α is a 157 amino acids 17-kDa protein having the gene on chromosome 6. Two soluble receptors (TNFR) are the main modality to control the activity, and receptor 2 has a five times higher affinity than receptor 1; nevertheless, TNFR-1 is responsible for the initiation of the majority of activities. That is because TNFR-1 is expressed on almost all cells, while TNFR-2 is only on immune cells.

TNFR-1 is the one responsible mainly for the apoptotic effect, being a member of the death receptor family. Yet, it can also induce cell survival signals [21].

TNFR-2 triggers the activation of nuclear factor kappa B. That induces the transcription of antiapoptotic, proliferative, immunomodulatory, and inflammatory genes, NF-κB being one of the most important survival factors in preventing TNF-induced apoptosis.

There is ample evidence of the role of TNF-α in the regulation of promotion and protumor damage, currently being defined as at least six characteristic aspects that provide the cells advantages of survival in vivo. These aspects promote cell-growth autocrine signals, installing insensitivity to anti-growth signals, angiogenesis favoring apoptosis pathways avoidance, tissue-invading, metastasis mechanisms favoring, and unrestricted cell replication [22,23].

Numerous studies prove the involvement of TNF-α in tumor promotion and propagation regulation. Thus, TNF-α favors NO production, which leads to other DNA alterations and cGMP-mediated tumor promotion. Equally, it favors cellular autocrine growth and is a survival factor for malignant cells (Table 1).

Some data suggest the upregulation of TNFR-1 on the membranes of endothelial cells within the tumor vessels, probably secondary to the presence of upregulating factors produced by vessel-surrounding cells (both tumor cells and macrophages). At least, in theory, that might mean that the tumor vessels are susceptible to anti-TNF-α treatments, so the biologic therapies have an antineoplastic effect [21].

Although many mechanisms make TNF-α a formidable favoring factor of tumor genesis, several mechanisms are included in the category of neoplasia counteracting. Thus, Umeno [24] proved that the intratumorally TNF-α injection leads to blood flow stops, with tumoral hemorrhages and vascular necrosis.

Moreover, the injection of TNF-α brings with it the important PMN tumor colonization, with a role in combating uncontrolled cell multiplication. Enabling macrophage cells and NK is another antitumor mechanism of α-TNF, as Blankenstein showed in 1991 (Table 2) [25].

Due to these complex and complementary effects that show that the body is the possessor of an extremely well-regulated balance in terms of its inflammatory antitumor defense mechanisms, an immunomodulatory treatment that influences the circulatory and tissue TNF-α concentrations can raise questions and concerns.

## 3. IL-12 Family

IL-12 family consists of IL-12, IL-23, -27, and -35, with important roles in the immune response mechanism of the skin. Of those, IL-23 is presently accepted as one of the most prominent inflammatory molecules in the development of psoriasis, being the target of some of the most modern biologic therapies.

The fundamental role is to act as the link between the innate and adaptative immune systems, with the development of CD4+ T cells into various helper T-cell subsets. Secreted by various cells in the skin (macrophages, dendritic cells), IL-12 and IL-23 are mainly proinflammatory cytokines, required for the development of Th1 and 17 cells [26]. Their balance may have a role in the development of anti- or pro-tumor immunity.

At this moment, it is considered that the main source of IL-23 is the myeloid cells after activation by both endogenous and exogenous signals. A variety of studies identified several such signals, such as tumor secreted factors (PGE2), damage-associated molecules, and pathogen-associated molecules [27,28,29,30]. As previously shown for α-TNF, the balance between IL-12 and IL-23 plays an important role in tumor initiation, growth, and progression, IL-12 mainly playing preventive roles and IL-23 promoting roles via the IL-17 family cytokines [31].

According to Cao et al. [32], IL-12 can modulate the tumor microenvironment to become more conducive to antitumor immunity. The main roles appear to be the inhibition of angiogenesis and the expansion of intratumoral Tregs. Nevertheless, it seems that IL-12 activates multiple pathways of antitumor immunity. At this date, there are strong arguments for believing that the antitumoral and antimetastatic roles of IL-12 are mediated via STAT4 activation of IFN-γ [33].

On the other side, IL-23 was found overexpressed in numerous cancers, such as non-small-cell lung carcinoma, colorectal carcinoma, hepatocellular carcinoma, ovarian, breast, pancreatic cancer, malignant melanoma, bladder cancer, and multiple myelomas, and gastric cancer [34]. The pathway is not yet clearly understood because although IL-23 is strongly linked to the cytokine production of Th17 lymphocytes, it is proven that it may have a tumor-promoting effect independent of IL-17. Kulig et al. [35] recently demonstrated that in the case of mice lacking IL-17A, reduced lung metastases are found, while mice lacking IL-23 were not showing the same aspect. Nevertheless, IL-17 is involved in the tumor-promoting mechanisms of IL-23.

A significant function of IL-23 seems to be the capacity for tumor metastases promotion via the up-regulation of the pro-angiogenic factors. The overexpression of IL-23 was strongly associated with metastasis in various tumors, such as hepatocellular and colorectal carcinomas, melanomas, and thyroid cancer [36,37,38]. In cases of human esophageal cancer, the overexpression of MMP9 and vascular endothelial growth factor (VEGF)-C was found to secondary facilitation of epithelial–mesenchymal transition and migration abilities [39].

All those studies suggest strong support for the involvement of IL-23 in the pathogeneses of different neoplasia, and mostly in those cases associated with an inflammatory supposed induction [40].

The drug-induced inhibition of IL-23, as resulted from the modern anti-psoriatic therapies, would therefore appear to be tumor protective.

The skin has a very important role in immunology, being involved in the maintenance of homeostasis of the body and tissue reparation. IL-23 is involved both in the etiopathogenesis of cutaneous diseases, the most significant example being psoriasis, and in the etiopathogenesis of other systemic diseases [41].

It has been proven that the level of IL-23 is increased on the level of tegument with psoriasiform lesions, unlike healthy skin [42]. The pathogenesis of the disease relies on the activation of dendritic cells in the derma of patients with psoriasis [43], which secrets high quantities of IL-23 [44]. Therefore, the increased expression of IL-23 maintains the inflammatory process characteristic of such a disease.

The efficient treatment of psoriasis determines the reduction of the level of IL-23, which proves the connection between the active disease and supra-production of IL-23.

IL-23 is involved in the occurrence of an inflammation on the level of the synovial membrane [45], and the application of anti-IL23 therapies enjoyed real success in psoriatic arthritis, determining a better quality of patients’ lives, up to complete remission in some patients [46].

The increased serum level of IL-23 was also identified in patients diagnosed with carcinomas; however, the role of such a molecule in the development of neoplasia is controversial [47].

## 4. IL-17

IL-17 main role may be defined as a stimulator of acute inflammation. It originated mainly in the CD4+ Th17 cells.

Those cells are most prevalent in bacterial and fungal infections, inducing inflammation that leads to the destruction of extracellular pathogens. They are considered today as the defining cell of type 3 Th immunity. The fundamental role is neutrophile recruitment and, in second, monocyte recruitment [48].

IL-17 stimulates cytokine production by other cells, leading to leukocyte recruitment. Probably, their involvement in psoriasis pathogenesis is correlated with the role of immune promotion and orchestrating [48].

The development of LyTh17 cells is controlled, at least in some part, by the Il-12 family cytokines, and that is the reason that IL-23 inhibitors result in a decrease in Il-17 levels.

The IL-17 is a family of six cytokines (from IL-17A to IL-17F) that acts on five receptors (from IL-17RA to IL-17RE). While IL-17 B, C, and D are mostly produced by epithelial cells, IL-17 A and F production span over several types of immune cells; those two subsets seem to be more connected to psoriasis than others.

The IL-17A cytokine’s role is to promote the production of neutrophils, being a strong chemotactic factor (as for monocytes). IL-17F, on the other side, is connected to the production of antimicrobial peptides, secretion of epithelial-integrity-related claudins, and production of inflammatory cytokines [49].

The IL-17 cytokines are not potent inflammatory molecules by themselves; their ability is mostly via the recruitment of cells in synergy with other proinflammatory cytokines (TNF, IL-1β, IFN-γ, GM-CSF, and IL-22) [49].

IL-17 is proven to induce neo-angiogenesis through activation of the vascular endothelial growth (VEGF) factor pathway and to enhance the activity of matrix metalloproteinase-2 and -9 (MMP-2 and MMP-9), which increase the metastatic activity of the underlying malignancy [50].

IL-17 does not seem to act as a survival factor for epithelial cells; nevertheless, it suppressed apoptosis of several tumor cell lines in vitro, suggesting a potential to promote tumorigenesis directly [50].

Kryczek et al. showed that both IL-17+ CD4+ and IL-17+ CD8+ T cells are present in the tumor microenvironment of different human tumor types, suggesting that local production of IL-17 by tumor-infiltrating T cells may be a relatively widespread phenomenon in tumorigenesis [51].

Karpisheh et al. consider that the direct effect of IL-17 on tumor cell survival predominates over other mechanisms; they proved that the blockade of IL-17 response in the studied tumor cell was as effective as CD8+ cell depletion in suppressing tumorigenesis. However, the contributions of IL-17 effects on tumorigenesis are likely to vary between tumor types, depending on the ability of the tumor cell to use IL-17 as a pro-survival factor [52].

Most recent studies also demonstrated that inflammatory responses can promote tumorigenesis via several different mechanisms, including pro-proliferative effects on the tumor, enhanced angiogenesis, and suppression of cytotoxic T-cell responses [53]. Inhibition of cytokine production could be a potential treatment both for chronic inflammatory diseases and tumor modulation.

The development of IL-17A inhibitors was a major milestone in psoriasis therapy, either alone (Secukinumab and Brodalumab) or in association with IL-17F (Ixekizumab). It is at this moment proven that those molecules can reverse clinical, histologic, and molecular features of psoriasis, superior to TNF-alpha inhibitors [49].

The potential efficacy of IL-17 inhibitors in the treatment of other dermatologic conditions (such as lichen planus, pyoderma gangrenosum, pemphigus vulgaris, pemphigoid, or dermatitis herpetiformis) is something that remains to be proven in the following years.

## 5. Discussion

The last decades of research and clinical experience in psoriasis have switched our understanding of the pathogenesis of inflammatory skin diseases, mostly psoriasis vulgaris, which is no longer regarded as a disorder primary by keratinocytes hyperproliferation. The current view is that of a genetically programmed pathologic interaction between resident skin cells, infiltrating immunologic cells, and proinflammatory cytokines, chemokines, and growth factors produced by these cells.

Today we are aware that psoriasis is an immune-mediated disorder in which intralesional T lymphocytes—via their proinflammatory signals—trigger and maintain rapid proliferation of basal keratinocytes, but we do not understand which is the primary stimulus that incites this inflammatory cascade. We know some of them, but only a few, and the exact mechanism involved is not well understood.

The demonstrated involvement of immune cascades is the basis for modern therapies. The most widespread psoriasis biologic treatments are:

- TNF-α inhibitors (Infliximab, Etanercept, Adalimumab, Golimumab, Certolizumab)
- IL12/23 inhibitors (Ustekinumab)
- IL23 inhibitors (Tildrakizumab, Guselkumab, Risankizumab)
- IL17 inhibitors (Secukinumab, Ixekizumab, Brodalumab)

Probably, they represent the most important breakthrough in psoriasis therapy since the discovery of topical anti-inflammatory molecules. While not curing the disease, biologicals offer a real consistent option for long-time control of clinical manifestations and a non-progressive course in arthritis. In very few words, the biologicals are targeting the activity of both the immune cells and their cytokines, resulting in four main mechanisms of action: reduction of number and activity of pathogenic T cells, reduction of activation (recruitment) of new T cells, immune deviation from a Th1 type immune response to a Th2 type response, and blocking the activity of involved over-expressed cytokines.

The TNF inhibitors are, at this moment, well-known therapeutic options, with plenty of information regarding their ups and downs, so a discussion about them is probably not necessary. On the other side, the IL-23 and IL-17 inhibitors are relatively still new.

Ustekinumab is a humanized monoclonal antibody, the type IgG1 kappa (IgG1κ), that antagonizes IL-23. This molecule can also inhibit IL-12. These two interleukins have an important role in the modulation of the lymphocytic Th1 and Th17 activity. This monoclonal antibody binds to the p40 subunit, which is found in the structure of IL-23 but also in the structure of interleukin 12, preventing them from attaching to specific receptors. By preventing IL-23 from attaching to receptors, Ustekinumab inhibits intracellular phosphorylation and production of other interleukins, such as IL-17A, IL-17F, and IL-22. Therefore, this monoclonal antibody blocks intracellular signaling and synthesis of other cytokines that depend on the IL-23 action [54].

Guselkumab is another humanized immunoglobulin, IgG lambda type (IgG1λ), that has anti-IL-23 activity. This molecule binds to the p19 subunit of the IL-23, thus blocking the attaching of this interleukin to its specific receptors. It has been shown that in some autoimmune diseases, including psoriasis vulgaris, there is an overexpression of both p19 and p40 subunits of the IL-23 [55]. By blocking the activity of this interleukin, Guselkumab inhibits the aberrant immune response that leads to uncontrolled hyperproliferation of keratinocytes and the development of psoriasis plaque [56,57].

Another effective therapy for the treatment of moderate to severe psoriasis vulgaris in adult patients that also has anti-IL-23 activity is Risankizumab, a completely humanized monoclonal antibody, IgG1 type. This molecule binds with high affinity to the p19 subunit of the IL-23. In this way, it blocks the attaching of IL-23 to specific receptors [58].

Tildrakizumab is another molecule with action against IL-23, a humanized monoclonal antibody of the IgG1 lambda (IgG1λ) type used in the therapy of moderate to severe psoriasis vulgaris in adults and which in studies has shown efficacy in patients with psoriatic arthropathy. Anti-IL-23 activity occurs by binding to the p19 subunit, thus preventing IL-23 from interacting with its specific receptors [59].

Molecules directed against IL-23 have several therapeutic effects because the molecule they block plays a key role in the cascade of pathophysiological events in many diseases with an autoimmune mechanism. By blocking the immune and inflammatory response resulting from inhibition of IL-23 activity, the molecules listed above can produce several side effects. Among the most commonly reported side effects of anti-IL-23 therapy are infections (the most common of which are upper respiratory tract viral infections, the incidence of severe infections being below 1.5%), the risk of developing malignancies, headache, arthralgia, myalgia, injection site reactions (erythema), odynophagia, cough, diarrhea, nausea, vomiting, fever, chills, anaphylactic reactions, pruritus, increased liver enzymes (7.43% of patients), cardiovascular events, hypertension arterial, purpura, paradoxical reaction (worsening of the underlying pathology), and depression [60].

It seems that molecules with anti-IL-12/23 action (Ustekinumab) appear to be associated with a higher incidence of adverse reactions than therapeutic agents that strictly block the action of interleukin 23. Even the rate of neoplasms is higher during the treatment with anti-IL-12/23 versus anti-IL-23 therapy [60].

However, although the list of side effects that may occur during treatment is very long, biological therapies with anti-IL-23 mechanisms are generally well tolerated by patients, and the safety profile of these molecules is good [61].

Among the contraindications of biological therapy against interleukin 23 (IL-23) are hypersensitivity and severe active infections. Regarding the use of anti-IL-23 therapy in pregnancy, there are little data on the safety of these molecules in pregnant women, with Certolizumab (anti-TNF agent) remaining the molecule with the best pregnancy safety profile. However, there are more and more studies that show the possible involvement of IL-23 in the production of recurrent miscarriage and the beneficial effects that therapeutic blockade of this interleukin could have [62,63].

In patients with congestive heart failure, the European Guidelines for the Treatment of Psoriasis Vulgaris recommend the use of anti-IL-23 molecules instead of anti-TNF therapy [64].

Ixekizumab is an IgG4 monoclonal antibody that binds with high affinity and specificity to both IL-17A and IL-17A/F. Neutralization of IL-17A by Ixekizumab inhibits the proliferation and activation of keratinocytes, as well as the inflammation that causes the appearance of bone erosions and the pathological process of formation of new bone tissue. Ixekizumab does not bind to ligands IL-17B, IL-17C, IL-17D, IL-17E, or IL-17F ligands. In vitro binding tests have confirmed that Ixekizumab does not bind to human receptors Fcγ I, IIa, and IIIa or to the receptor for complement C1q.

Secukinumab is a fully human IgG1/κ monoclonal antibody that selectively binds to and neutralizes IL-17A. It works by targeting IL-17A and inhibiting its interaction with the IL-17 receptor, expressed in various cell types, including keratinocytes. As a result, Secukinumab inhibits the release of proinflammatory cytokines and reduces IL-17A-mediated contributions to inflammatory diseases.

Brodalumab is a human monoclonal IgG2 antibody directed against human IL-17RA. It is expressed in a Chinese Hamster Ovary (CHO) cell line. Brodalumab is comprised of 1312 amino acids and has an estimated molecular mass of 144,000 Daltons.

We are constantly discussing evidence-based medicine in this context as well, and, just as we suggested above, both the clinical trials conducted so far and the meta-analyses, the data from international registers, and, last but not least, the clinical experience of specialists are the powerful tools that we have at hand when we make the claim that so far there are no data to show that anti-TNF-α and anti-IL-23 therapies would be directly criminalized in the induction of neoplasia, be they organ or hematological.

However, it would be troublesome not to admit that psoriasis biological therapies, like all therapeutic formulas with a degree of immunosuppression, might, in theory, favor the development of neoplasia, either pre-existing or de novo. By disturbing the cytokine balance, the anti-TNF-α and anti-IL-23 biological therapies can alter in an unpredictable measure the immune surveillance of the organism over some neoplasia existing at the time of treatment initiation but insufficiently developed to be detected with the currently available methods.

Vigilant post-marketing supervision of medicinal products is vital due to the circumstances of the clinical trials currently in use. Thus, for the introduction of new medicinal formulas on the market, in human therapy, it is well known the need to prove both the effectiveness of the products and the absence of risks. Nevertheless, phase 1, 2, and 3 clinical trials are studies conducted on a small number of human subjects (several hundred or thousands), and the duration of treatment monitored in studies is relatively short.

Thus, for treatments that are suggested to be followed throughout life, such as anti-TNF-α and anti-IL-23 products, studies have followed the safety over 4–5 years maximum, data beyond this range being collected exclusively from national registers or case communications.

In addition, the selection of patients enrolled in clinical trials is extremely rigorous, thereby rendering an imbalance that cannot be ignored between the research data communicated and daily practice. Patients in clinical trials are selected in a manner by which we could call them “healthy patients”, with minimal pathological associations, without drug interactions, and often in a well-controlled environment. This leads to the situation that in current practice, the use of drugs is allowed, including in categories of patients who have been excluded from clinical trials and against which safety data are incomplete.

Meta-analyses replace a number of these clinical trials’ disadvantages, in the first row the limited number of patients. Duration of individual exposure to active molecules, however, does not change, although the data are communicated in the statistical form of patient-years type exposure.

In addition to this, a meta-analysis of nine clinical trials [65] shows a three times higher risk (OR 3.3 with CI 95%) of developing a malignancy in patients treated with anti-TNF-α vs. control; the risk is proportional to the dose administered. Nota bene, in the mentioned study, the rate of neoplasia in the control arm was abnormally low [65].

## 6. Conclusions

The systemic inflammatory syndrome concept is one of the foundations that stand at the basis of revolutionary modern and future therapies. It stands on the in-depth understanding of the delicate mechanisms that govern the collaboration between the systems and organs of the human body and the fine balance that ensures a reproach-free operation. The relatively recent discovery of the intricate and complex role of cytokines in the pathophysiology of various diseases (previously considered locally determined and manifested) shaped a new understanding and approach to so many diseases, psoriasis being only one of them.

The environment-inadequacy status is a concept that we propose, one that incorporates all the situations of the organism’s response disorders (be they immune or hormonal or otherwise) in the face of imprecisely defined situations of the environment. As long as we do not know the specific (eventual) trigger factor of inflammation in different situations, we will probably do only minor steps in our understanding. Furthermore, our understanding will probably be based on experiments and pharmacologic breakthroughs.

The correlation between these two concepts will likely shape the future of modern medicine, along with the gene-adjustment mechanisms.

Psoriasis is a clear example of an inadequate body response as a result of exposure to as yet undefined triggers with an excessive systemic inflammatory reaction, insufficiently controllable. The discovery of biological therapies has been a real success. Their use in autoimmune diseases such as psoriasis (and psoriatic arthritis) has improved the quality of life of patients, and therapies targeting those interleukins could be a new treatment strategy in other autoimmune diseases as well.

Because of the important roles of cytokines in the control of cancer promotions and control, concern was raised about the fact that the use of biologicals may alter immune surveillance and promote cancer progression. Both theoretical and practical data nevertheless showed that, in fact, the treatment-induced control of cytokines may be beneficial, reducing the inflammatory milieu that promotes cancer.

Biological therapy is extremely useful and brings spectacular clinical results with a proper safety profile. Yet, we must bear in mind that patients eligible for biological therapy should be correctly and carefully evaluated previously. Any patient on the biological treatment with even minimal changes in biological constants requires thorough evaluation. There are no “trivial” or “insignificant” alarm signs; actually, alarm signs are by definition alarming and worrisome, so they must be taken into proper consideration.

## Figures and Tables

**Table 1:** TNF-α roles in the regulation of tumor promotion and propagation [17,20,21,24,25].

TNF-α Roles
NO production
Autocrine growth and survival factor for malignant cells
Tissue remodeling by matrix metalloproteinases
Control of leukocyte infiltration by cytokine modulation
Inhibition of E-cadherin, nucleolar b catechin growths
Increased tumor motility (invasiveness)
Epithelial–mesenchymal transition
Induction of angiogenic factors
Loss of androgen response
Resistance to cytotoxic medication

**Table 2:** TNF-α roles in combating neoplasia [17,20,21,24,25].

TNF-α Roles
Blood flow stops, intratumoral bleeding, vascular necrosis (intratumoral injection)
Tumor colonization with PMN
Inhibition of tumor growth by macrophages and NK cells
NK- and LAK-induced destruction and tumor rejection
Antitumor immunity and tumor-induced CTL removal
Favoring cytochrome c mitochondrial induced apoptosis
c-myc-dependent apoptosis
Inhibition of NF-kB activation

## Data Availability

Not applicable.
